# A Practical Example of GaN-LED Failure Cause Analysis by Application of Combined Electron Microscopy Techniques

**DOI:** 10.3390/ma10101202

**Published:** 2017-10-19

**Authors:** Elke Meissner, Maral Haeckel, Jochen Friedrich

**Affiliations:** 1 Fraunhofer Institute for Integrated Systems and Device Technology, Schottkystr. 10, 91058 Erlangen, Germany; jochen.friedrich@iisb.fraunhofer.de; 2 SiCrystal AG, Thurn-und-Taxis Str. 20, 90411 Nuremberg, Germany; maral.haeckel@sicrystal.de

**Keywords:** GaN, LED, failure, EBIC

## Abstract

In this paper, we report a failure case of blue LEDs returned from a field application, and propose a practical way to identify the physical and structural reasons for the observed malfunction by a combination of different electron microscope techniques. Cathodoluminescence imaging and electron beam induced current (EBIC) imaging are employed in order to visualize conductive paths through the device in conjunction with subsequent energy dispersive x-ray analysis (EDS), revealing a metal deposition along cracks in the semiconductor layer which short-circuit the device. We demonstrate that the electron beam induced current imaging, in conjunction with other microscopic and analytical techniques at µm scale, is a powerful combination for clearly resolving and visualizing the cause of failure in the GaN LED chip. However, this represents a case study of a real application, which may not have been generally observed in laboratory testing environment.

## 1. Introduction

GaN-based white and blue LEDs are becoming more and more attractive, not only for general lighting applications or the use of LEDs in general or car lights, but also more and more widely for diverse purposes in industrial environments. In such environments, the devices be subject to various environmental conditions, e.g., mechanical influences such as vibrations, enhanced or extremely low temperatures, humidity, chemical substances, and others. Although there is a specification given for the operating conditions of a device, it is unclear how a device may react with regard to its long-term stability, since experiences with this type of device in long-term field applications are still few. Moreover, if a malfunction of a module or system occurs, it is not necessarily clear whether the LED chip itself, or a peripheral element, has caused the failure. Very recent publications [1] indicate that the chip itself may be quite stable, but other components, like optical elements, phosphors etc., may not be. Moreover, there are indeed cases where the LED chip has broken. From the first observation of malfunction of a LED, it is not always unambiguously clear which of these cases, in fact, applies. Most examinations of LED chip failures have been based on electrical analysis and stressing in order to, for example, define specifications or save operating conditions for a device, or to predict its lifetime, or to gain knowledge about aging mechanisms of an LED in a system [2,3]. On the other hand, there have been investigations reported in the literature related to the physical cause of device failure due to material issues inside the LED, which are quite sophisticated [4]. It is simply not possible to test or evaluate any type of potential environmental influence, or combination thereof, that could possibly appear during operation prior to a customer’s usage. Therefore, laboratory testing procedures don’t comprise all possible cases, and there may be cases in real field application that were not foreseen by experimental testing. Hence, the physical reasons why an LED, mounted in some application in the field, fails are numerous, and may be distinct from the causes found during quality control or standard laboratory testing carried at the chip’s production site. Thereby, the reliability of devices must be regarded not only in terms of their physical lifetime under laboratory test conditions, but also with regard to a customer’s expectations of lifetime within a system without malfunction [5].

It is therefore necessary to define a practical route for identifying the physical reasons for device failure of a LED directly after being returned from field application by a customer. Analytical techniques applied for this should be capable of observing the LED structure on a macroscopic as well as on a microscopic scale, structurally as well as electrically. There are numerous examples of systematic reliability studies in the literature, which were already frequently being performed on GaAs-based or other classical LED structures quite a time ago, e.g., [6] and others. GaN-based LEDs have been studied intensively in recent years in order to better understand their degradation mechanisms and their countermeasures. However, the majority of investigations have dealt with the appearance of failure pictures depending on parameters like, e.g., overvoltage, current density variations, temperature changes, thermal cycles, etc., tested in laboratory environments. A decent description may be found, e.g., in [7].

In this study, we describe the analysis of blue light LEDs that had been used in a harsh environment. The LEDs were returned from the field after exhibiting various malfunctions, which will be explained in the later sections, with unclear origins. We would like to point out the practical aspect of this study, and the potential of electron beam-induced current (EBIC) imaging in a scanning electron microscope (SEM) for the identification of potentially present electrically conductive paths, which could short-circuit the device in this case. The SEM in parallel offers the possibility of directly observing the same place previously electrically characterized with regard to its structural constitution. Moreover, it was possible to carry out energy dispersive x-ray analysis (EDS) at that particular location on a µm- or sub-µm scale in order to observe possible changes in the elemental composition of structural elements of the LED chip or displacements of elements through migration in the structure. This paper aims to demonstrate the strength of this correlative analysis route for the identification of reasons for unexpectedly short lifetimes in LEDs operated in industrial applications.

## 2. Experimental Approach

The investigated LEDs we received had been mounted in modules with varying numbers of single devices of differently colored LEDs. The modules stemmed, e.g., from an application where the LEDs were used for illumination in industrial washing systems. The devices in these systems were exposed to an environment that was humid, as well as variable in temperature, and they were exposed to chemical contamination. The devices were, of course, applied within the given specifications regarding temperature and electrical operation conditions, and were sealed against the surrounding atmosphere by encapsulation. Therefore, it was expected that the LEDs should survive the specified lifetime of many thousand hours, functioning correctly. However, after a short time, the blue and white LEDs either started to flicker, showed a reduced light output, or spontaneously failed completely, whereas the red LEDs present in the same module in parallel continued to function correctly. After continued operation of the modules, the total number of failed devices continued to grow, until an unacceptable status was reached. First, it was suspected that, during the fabrication of the LED modules, some electrical discharge events occurred, which could have damaged the blue and white LED chips, in particular. Consequently, it was carefully proven through the full fabrication process that this was not the case. The true cause of failure was thus still unknown.

**Figure 1:**
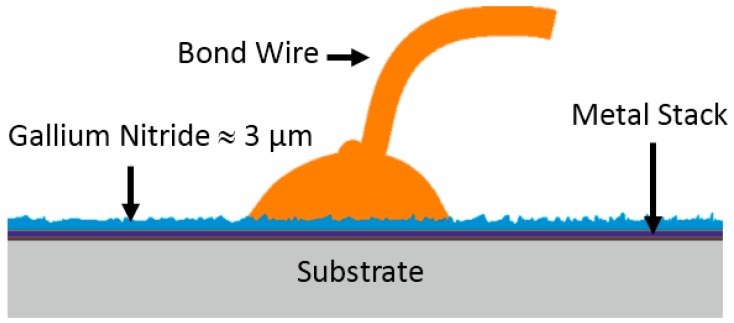
Schematic drawing of a blue LED chip inside the SMD device. The functioning nitride layer is mounted on a substrate through a metal solder containing different elements. The surface of the nitride is rough, and the bond ball is placed on top, where the bond wire forms the contact with the bond ball.

The LEDs were common SMD-type devices (surface mounted devices) with a conventional sealing, including a converting phosphor. The electrical contacts are backside, and the devices were, as mentioned before, mounted and operated following the given specifications. The LEDs were grouped into two cases based on their failure behavior. The first group collects those LEDs that failed directly after switching the device on (spontaneous failure). The second group consists of the LEDs that failed after a certain run time in the field application, referred to as “field failure”. The field failure cases were subdivided further into four typical cases: (A) the LED showed a reduced light output; (B) the LED flickered between dark and bright; (C) the LED switched between on and off; (D) the LED did not emit light any more. With this in mind, one could think of a number of different failure causes. Figure 1 depicts a basic schematic drawing of the blue LED chip used inside the devices. The substrate is a bond wafer, and the metal stack contains the common metals expected for contacts in GaN LEDs.

The SMD devices were, first of all, carefully inspected for visible damage. The packages did not show any cracks, and there were no color changes of the phosphor visible to the naked eye, and no deformations of the package or anything else. Thus, after a thorough first inspection, one could finally conclude that the cause of failure was not due to obvious chemical or thermal degradation, since there were no hints found in support of that.

The same applied for the backside contacts, and the bond wires and feet. The front side and backside contacts were additionally electrically tested, and were found to be intact. No failure or degradation of electrical contacts could be identified.

After that, the prepared LED chips, now free of any coverings, were subjected to cathodoluminescence (CL) imaging at a Jeol 7500F SEM (JEOL, Tokyo, Japan) equipped with a Gatan CL3+ system and spectrometer. The CL images shown in this paper are panchromatic recordings using primary beam energies of the microscope of 5–10 kV. The cathodoluminescence imaging of GaN as a material can help to visualize areas of structural damage where, as a result, a reduced luminescence would be expected. The GaN emits light under electron-beam irradiation, whilst remaining dark at those places where the material has been physically or structurally disrupted, thus creating a visible contrast against the surroundings. Typical damage caused by an electrical overstress or discharge event could cause damage such as those described, e.g., in [8]. Normally, one would expect that these events would cause small, flat “surface craters”, which would appear dark in the CL image due to the structural destruction of the material. Therefore, CL imaging can be regarded as an easy and reliable method for identifying structurally disturbed areas following electrical stress testing and inspection for unexpected discharge events.

**Figure 2:**
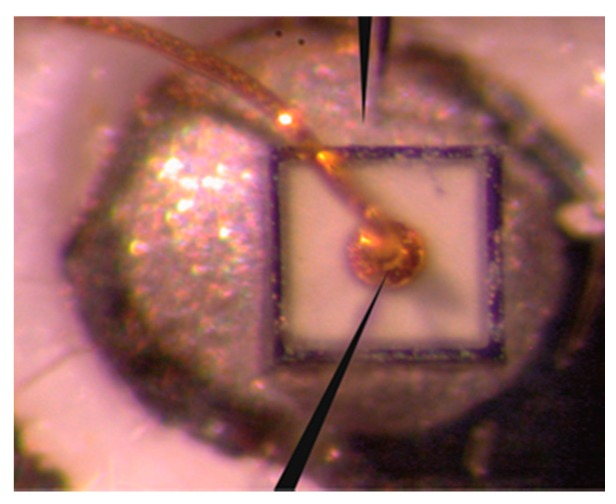
The blue LED chip prepared for the EBIC analysis. One can see the chip with the bond foot and wire. The chip is contacted by the EBIC tips (arrows). The side length of the chip is approximately 0.3 mm. The coverings were removed by chemical means.

For further investigation, we prepared cross-sections of the LED chips. Of course, it is necessary to ensure that issues observed in later imaging are not e result of mechanical treatment. Moreover, we applied electron beam-induced current (EBIC) imaging to these cross-sections to identify local differences in current flow. EBIC was performed with a SEM Jeol 6610 (JEOL, Tokyo, Japan) using a Kleindieck 4-tip prober shuttle (Kleindieck, Reutlingen, Germany). The electron beam’s interaction with the sample induces excess carriers, which are separated in an external field or at a space charge region. At those places where the current flow is disturbed, e.g., by a structural disruption, the carriers will recombine, and thus no EBIC current will appear. The EBIC current is encoded in grayscale, and an image of current flow can therefore be generated. At places of high conductivity, a bright contrast is expected to appear. At the same time, a SEM picture can be collected, such that probable morphological features can be confidently identified. Therefore, failed and intact devices were investigated in this way. Figure 2 shows an example of an LED prepared for EBIC investigation.

One can see the LED chip and the bond, as well as the two EBIC tips in contact with the device. In summary, the above-mentioned route of investigation through combined analytical techniques was capable of clarifying the failure cause of the LEDs, as will be further discussed in the results section, below.

## 3. Results and Discussion

**Figure 3:**
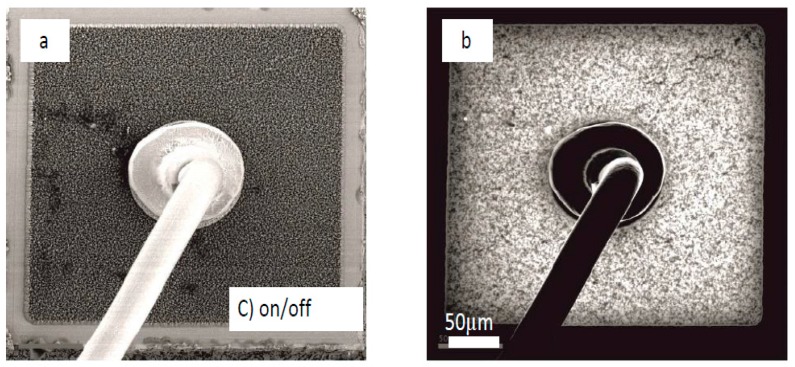

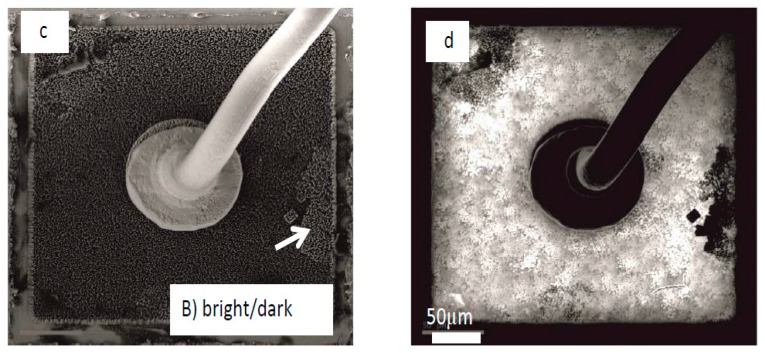
Cathodoluminescence imaging of case (B) and (C). On the left side, the SEM pictures are shown, illustrating that no surface defects can be found. There are only some cleaning residues visible (arrow), which could obviously not be removed during the preparation of the free chip surface. (**a**) SEM image of failure case (B); (**b**) Corresponding CL image of case (B); (**c**) SEM image of failure case (C); (**d**) Corresponding CL image of case (C).

Figure 3 shows the panchromatic CL image of LED chips of the (B) (dark-bright) and (C) (on-off) cases under electron-beam irradiation. Usually, the surface of the nitride layer is roughened during the production process, so that better light extraction can be attained. For CL imaging, this causes the fine bright and darker “grainy” contrast in the picture. Aside from that, the nitride surface homogeneously emits light. The dark areas visible in Figure 3c,d are obviously residues (arrow) from surface cleaning (confirmed by corresponding regular SEM imaging), which could not be further removed.

Finally, nothing was found that could imply structural damage that could be related to ESD events for any of the failure cases described above, as can be seen from the figure. Wavelength selective imaging did not reveal any further information. This was the finding for all of the investigated devices, irrespective of failure type. In cases of discharge damage, material displacements would usually be clearly visible. However, depending on the level of impact, a chip may receive damage in the active zone, which would not be visible from the surface, but such case would presumably not correspond to the observed malfunction symptoms.

**Figure 4:**
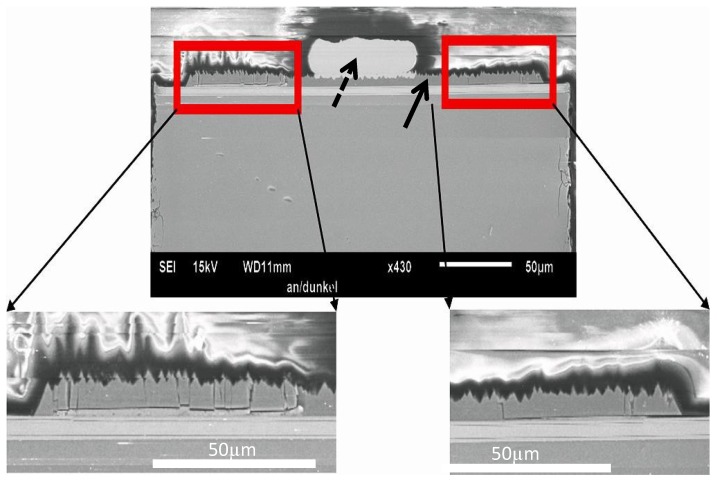
SEM cross-section picture of an example of a device with type (B) failure case (flickering dark and bright). One can see the bond foot (dashed arrow) and the nitride layer (solid arrow). The nitride layer has lost contact to the back side, and shows partial cracks, which are visible from the enlargements.

The further observation of cross-sections of the nitride layers inside revealed the reasons for the devices’ malfunctions. In Figure 4, an example of a cross-section of an LED exhibiting failure type (B) (flickering dark and bright) is shown. One can see that beside the bond foot, the nitride semiconductor layer has slightly lost contact to the back side of the device and, moreover, shows tiny cracks across the layer.

**Figure 5:**
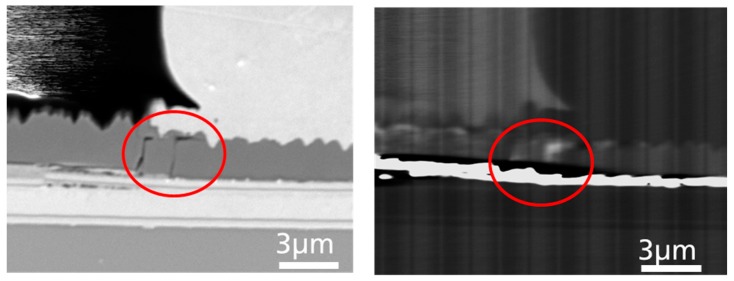
Left side: A crack is visible directly under the bond ball (red circle) in the SEM picture. Right side: The corresponding EBIC image shows a bright contrast at the crack position, indicating local conductivity. Such observation was associated with failure case (D).

Therefore, in that case, the failure symptoms (bright and dark) may appear because the nitride layer has only sporadic contact to the backside, and as such, functions electrically only partially. Hence, the device is not, in principle, fully broken, but the total area emitting light may change, resulting in a darker or brighter light output. This may, in the same way, also cause a LED not to flicker bright and dark, but simply to show a reduced light output due to the fact that a part of the semiconductor layer has broken off completely, and only the remaining material is emitting light. This is thought to explain the failure cases (A), (B) and (C). This suggestion is supported by the findings that were made on the field failure case type (D), LEDs which lost their functionality completely. Figure 5 depicts a SEM, as well as the corresponding EBIC image, from such a device in a cross-section picture.

Again, it can be seen that the nitride layer is broken, but in this case, directly underneath the bond foot. There are two cracks visible through the layer reaching from the backside to the bond. However, this may not be distinct from the above-mentioned case as long as it’s just the crack.

Therefore, in this case, the LED experienced a short-circuit. In order to distinguish why this case leads to a direct shorting of the LED, rather than to one of the other failure cases, it was suspected that a conductive path was involved between the front pad and the backside. Of course, a shorting of a device could be measured directly by an I-V measurement, but that wouldn’t deliver information about the physical or microscopic reasons for the short circuit. Microscopic imaging alone may show the microscopic features, but would not raise the fact of whether a particular feature was conductive or not, which could lead to misinterpretation, since the conclusions would be indirect. Therefore, EBIC imaging was applied, since that method is capable of imaging microscopic issues in parallel with its electrical properties. The EBIC current measured was thereby converted into a grayscale image. Bright contrast is related to a high EBIC current, i.e., to high conductivity. The corresponding EBIC image in Figure 5 clearly reveals that one of the cracks is highly conductive (bright contrast), vertically short-circuiting the device from the backside to the bond. The other crack does not show the same bright contrast, which means that there is no current flowing. This could be due to a different contact between that particular feature and the backside and bond, even though it may have the same properties; or, alternatively, it does not have the same conducting properties. The latter is more unlikely, from the following EDS results.

**Figure 6:**
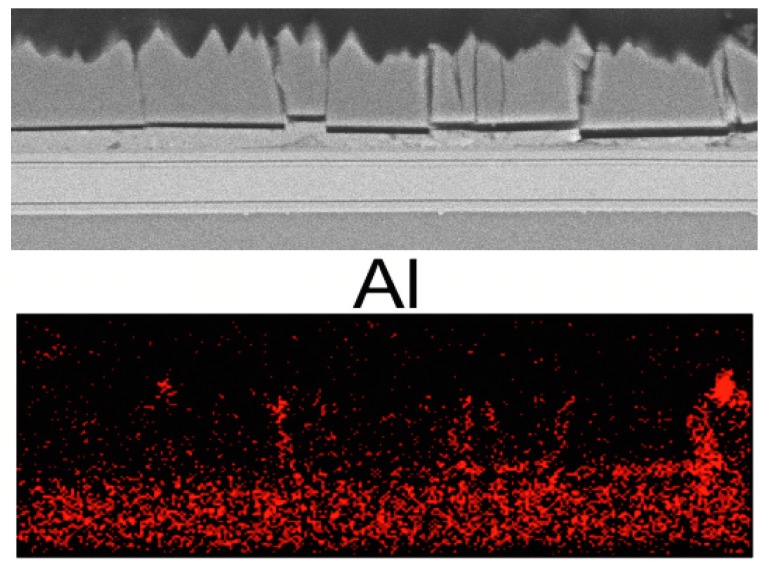
Top figure: SEM picture, showing a region with cracks located close near the bond. Bottom figure: EDS mapping at the same place shows the Al-EDS signal, which images the crack locations. The total width of the picture is 50 µm.

In order to clarify why this crack is conductive, in contrast to the others seen before, which were not vertically conductive and did not short-circuit the device, a subsequent EDS element mapping of an area exhibiting cracks in the semiconductor layer close to the bond was collected (Figure 6). The situation shown in Figure 6 corresponds to the case (D) depicted in Figure 5 above.

This crack could be identified as containing aluminum. The crack apparently offered a path for the metal atoms to move from the backside metallization through the cracks upon electrical operation of the device, and by this means short-circuit the device at a later point in time, which is thought to explain the failure case (D).

However, the reason for the metal moving along the cracks in this case is not obvious, as the LEDs were operated in line with the producer’s specifications during their application in the modules. The phenomenon of electromigration is usually expected under large currents or elevated temperatures [7], conditions which can be excluded for the application case.

Beside the bare observation as to what the physical reason of the failure of the LEDs was, there remains, of course, the question as to why this happened during regular operation of the devices within the specifications. There have been numerous failure scenarios described in the literature that were found after advanced and intensive testing of devices (see also many references in [8,9]). However, a “field return case” does not typically offer the possibility of interpreting the findings on the basis of systematic dependencies in operation conditions for a device, such as, e.g., current variations, systematic changes of temperature, etc., which, of course, make conclusions regarding the reason for the failure, and how to avoid the problem, much more difficult, and sometimes speculative.

## 4. Conclusions

This paper demonstrates a practical method for the evaluation of failure causes in light-emitting devices that have been returned from the field after malfunction, by means of a combination of electron microscopic and analytical techniques, including EBIC imaging. By these means, it could subsequently be shown that the reasons for the failure behaviors observed amongst the devices brought back were various. The approach is capable of explaining the different failure symptoms by identifying the various types of physical damage to the semiconductor functional layer. The strength of this approach is the direct correlation of electrical, chemical, optical and structural observations on the same sample, which helps to clarify even difficult and diverse failure symptoms. We believe that this route can be applied not only to optoelectronic, but also to other types of electronic devices, where the identification of the real physical cause of a failure is desired. However, more complex heterostructures may demand further or advanced preparation or sample treatments, as well as modified combinations of the techniques shown.
